# Combined Oral Contraceptive Use and the Risk of Cervical Cancer: Literature Review

**DOI:** 10.1055/s-0043-1776403

**Published:** 2023-12-23

**Authors:** Adriane Cristina Bovo, Priscila Grecca Pedrão, Yasmin Medeiros Guimarães, Luani Rezende Godoy, Júlio César Possati Resende, Adhemar Longatto-Filho, Ricardo dos Reis

**Affiliations:** 1 Hospital do Câncer de Barretos, Barretos, São Paulo, Brazil.; 2 Faculdade de Medicia, Universidade de São Paulo, São Paulo, SP, Brazil.; 3 Life and Health Sciences Research Institute, Faculdade de Medicina, Universidade do Minho, Braga, Portugal.; 4 Government Associate Laboratory, Braga, Portugal.

**Keywords:** Oral hormonal contraceptives, Cervical cancer, HPV, Contraceptivos hormonais orais, Câncer cervical, HPV

## Abstract

Cervical cancer (CC) is caused by persistent infection of human papillomavirus of high oncogenic risk (hr-HPV); however, several cofactors are important in its carcinogenesis, such as smoking, multiparity, and prolonged use of oral hormonal contraceptives (COCs). Worldwide, 16% of women use COCs, whereas in Brazil this rate is of ∼ 30%. The safety and adverse effects of COCs are widely discussed in the literature, including the increase in carcinogenic risk. Due to the existence of several drugs, combinations, and dosages of COCs, it is hard to have uniform information in epidemiological studies. Our objective was to perform a narrative review on the role of COCs use in the carcinogenesis of cervical cancer. Several populational studies have suggested an increase in the incidence of cervical cancer for those who have used COCs for > 5 years, but other available studies reach controversial and contradictory results regarding the action of COCs in the development of CC.

## Resumo

O câncer cervical (CC) é causado pela infecção persistente pelo papilomavírus humano de alto risco oncogênico (hr-HPV); entretanto, vários cofatores são importantes na sua carcinogênese, como tabagismo, multiparidade e uso prolongado de contraceptivos hormonais orais (COCs). No mundo, 16% das mulheres usam AOCs, enquanto no Brasil essa taxa é de ∼ 30%. A segurança e os efeitos adversos dos COCs são amplamente discutidos na literatura, incluindo o aumento do risco carcinogênico. Devido à existência de várias drogas, combinações e dosagens de COCs, é difícil ter informações uniformes em estudos epidemiológicos. Nosso objetivo foi realizar uma revisão narrativa sobre o papel do uso de COCs na carcinogênese do câncer cervical. Vários estudos populacionais têm sugerido aumento da incidência de câncer de colo uterino para aquelas que usam COCs há mais de 5 anos, mas outros estudos disponíveis chegam a resultados controversos e contraditórios quanto à ação dos COCs no desenvolvimento do CCU.

## Introduction

Cervical cancer (CC) is considered a malignant neoplasm with great potential for prevention and early detection. Worldwide, it is the fourth type of cancer in incidence and mortality among women, with an estimated 604,000 new cases and 342,000 deaths in 2020. More than 85% of these deaths occur in low- and middle-income countries, such as Brazil. 1 In the Brazilian population, it represents the third malignant neoplasm for women, and the National Cancer Institute (INCA, in the Portuguese acronym) estimates the occurrence of 17,010 new cases for the three-year period from 2023 to 2025 and the risk of 15,38 new cases for every 100,000 women, with 6,627 deaths recorded in 2020. 2

Persistent human papillomavirus (HPV) infection is a necessary but not sufficient cause for the development of CC. Types 16, 18, 31, 33, 35, 39, 45, 51, 52, 56, 58, and 59 are considered of high oncogenic risk (hr-HPV); thus, they are associated with high-grade intraepithelial and invasive cervical lesions. 3 Several other cofactors have an impact on the development of CC, including sexually transmitted infections, smoking, multiparity, and prolonged use of oral hormonal contraceptives (COCs). 4 5 6 7

After their introduction in the 1960s, COCs have revolutionized the reproductive lives of millions of women worldwide by allowing effective and convenient familiar planning. Since their wider use, a greater concern about potential adverse effects has motivated the emergence of new generations of COCs, combining lower estrogen doses with new and higher potency progestins and, consequently, fewer adverse effects. The benefit of familiar planning is not only limited to the fertility decline itself, but also to its socioeconomic impact due to maternal, newborn, and infant health benefits. Also, the indirect effects on women's education, income, and employment are relevant. 8 Nevertheless, according to a survey published in 2014, 40% of the 85 million pregnancies that occurred in the world in 2012 were unintended. 9

The widespread use of COCs is due to their well-established high efficacy and safety. According to data from the United Nations Department of Economic and Social Affairs, the COCs are the fourth most used contraceptive method in the world (∼ 151 million women), which represents 16% of the methods. In contrast, in Brazil, COCs are the most used method, representing 29.7% of the contraceptive choice. 10

Benefits and adverse effects of COCs are widely discussed, including the risk of cancer. 11 12 13 The association between the use of COCs and oncogenesis in the cervix is also quite controversial in the literature. Factors such as differences in the frequency of screening between users and nonusers of COCs and unreliable information are pointed out as confounding factors in the analysis. 14 In addition, the variability in active ingredients, combinations, and dosages in the presentations of COCs prevents epidemiological studies from having uniform information for consistent analysis.

A study analyzing the effect of COCs on three different patterns of incidence of genital cancer in several countries concluded that the use of COCs may contribute to the number of cases in countries with a high incidence of CC. 15 In Brazil, the wide use of the method demonstrates the need for a more careful evaluation of the likely impact of its use on the incidence of CC, especially due to the long-term use often seen among the Brazilian female population. Thus, the present article aims to perform a narrative review of the role of COCs use in CC carcinogenesis.

## Combined Oral Contraceptives and Carcinogenesis

The role of COCs in carcinogenesis has been discussed in the literature since the 1970s. 16 17 18 19 Estrogens and progestogens used for contraception and hormone replacement therapy have been also considered in the International Agency for Research on Cancer (IARC) study groups since the 1970s. In 1979, the IARC published a monograph with a general discussion on the subject, stating that steroid hormones are essential for growth differentiation and function of many tissues in animals and humans. Also, it was established by animal experimentation that modification of the hormonal environment by surgical removal of endocrine glands, by pregnancy, or by exogenous administration of steroids can increase or decrease the spontaneous occurrence of tumors. They also concluded that the incidence of tumors in humans can be altered by exposure to various exogenous hormones, individually or in combination, and that hormonal environment and dosage are involved in the carcinogenic effects of estrogens and progestogens. 20

Recently, in 2012, the IARC published data showing that the use of COCs may increase risks for some cancers and protect against others. Their use was associated with increased risks for breast cancer in women < 35 years old (both for current and recent users), carcinoma in situ and invasive carcinoma of the cervix, and even liver cancer in populations at low risk for hepatitis B virus infection. In addition, for CC, risks increased with the duration of use and decreased after discontinuation. For endometrial cancer, COCs had a protective effect that increased with the duration of use and remained at least 2 decades after discontinuation. There was evidence that the level of the protective effect was proportional to the progestogen potency of the preparation and inversely proportional to the estrogen potency. Concerning ovarian cancer, a greater risk reduction was observed with the duration of use and was persistent for at least 30 years after discontinuation. It was also suggested that COCs could reduce the risk of colorectal cancer and had the unlikely potential to change the cancer risk in the thyroid, lung, stomach, urinary tract, gallbladder, pancreas, lymph nodes, skin, and central nervous system. 21

Gierisch et al. published a meta-analysis confirming these findings. They included 44 studies of breast cancer, 12 of cervical cancers, 11 of colorectal cancers, and 9 of endometrial cancers. The incidence of breast cancer was slightly but significantly increased in female users of COCs (odds ratio [OR] = 1.08; 95% confidence interval [CI] = 1.00–1.17), with higher risk associated with recent use. The risk of CC increased with the duration of use in women with hr-HPV infection, but the heterogeneity of the studies prevented the meta-analysis on this topic. The incidences of colorectal cancer (OR = 0.86; 95%CI= 0.79–0.95) and endometrial cancer (OR = 0.57; 95%CI = 0.43–0.77) were significantly reduced by the use of COCs. 22 Similar results were described in a recent literature review published by Kamani et al., recommending that patients seeking family planning be advised of possible increased carcinogenic risk, but also advised of the advantages for sexual health and reduced risk of endometrial, colorectal, and ovarian cancer. 23

From the standpoint of population impact, a meta-analysis on estimated cancer risk in US women aged 20-54 years old using COCs was performed and showed that for every 100,000 women using COCs for 8 years, the estimated number of additional cases or fewer per 100,000 users is + 151 (breast), + 125 (cervix), - 197 (endometrium), - 193 (ovary), and + 41 (liver), concluding that for public health, the real effect is insignificant. 24

## Combined Oral Contraceptives and Cervical Cancer

Some experimental models have demonstrated the role of exogenous estrogens – such as those used in COCs – in cervical carcinogenesis. Experimental studies with transgenic mice expressing oncogenes for HPV 16 have shown that they rarely develop CC, although they can develop spontaneous skin tumors. However, with chronic exposure to exogenous 17 beta-estradiol combined with persistent oncogenic expression of HPV 16, it was demonstrated that benign epithelial hyperplasia acquired the ability to transform into neoplastic tissue in the cervix and vagina. 25 Also in transgenic mouse models, the removal of exogenous estrogen has been shown to lead to decreased progression or regression of pre-existing cervical neoplastic disease. 26 According to Chung et al. (2008), the estrogen alpha receptor (ER) is one present in the cervix. Transgenic mice with oncogenes for HPV 16 but deficient in ER had an inhibition of CC progression while exposed to exogenous estrogen, therefore proving the impact of exogenous estrogens on cervical carcinogenesis. 27

In a monography analyzing carcinogenic risk in humans of hormonal contraception and postmenopausal hormone therapy, the IARC listed some confounding and complicating factors for an accurate analysis of the relationship between COCs and CC. Firstly, women exposed to HPV infection are more susceptible to other sexually transmitted infections; thus, knowledge of sexual habits is necessary for correct analysis. In addition, it would be relevant to determine the persistence of hr-HPV infection, considering the hypothesis that COCs could increase the likelihood of the infection becoming persistent; a fact often ignored in studies at the time. It was also mentioned that ethical issues prevented large prospective studies with follow-up for women with HPV infection until the development of CC to assess the influence of COCs use. Finally, the bias of screening was pointed out, in which patients who use COCs undergo screening tests more often than nonusers. In conclusion, the monography analyzed 5 cohort studies and 16 case-control studies that showed a small increase in the relative risk of CC associated with the long-term use of COCs. This association was also observed in studies with analyses restricted to case-control studies with data on HPV infection and biases related to sexual behavior, screening, and other factors that could not be ruled out as possible explanations for the observed associations. 28

A possible mechanism to explain the association between the use of COCs and the risk of CC would be the possible interaction between estrogens, progestogens, and hormone receptors in cervical tissue, influencing the natural history of HPV infection. It is postulated that sexual hormones can potentiate the expression of E6 and E7 oncogenes of HPV 16, stimulating the degradation of p53 tumor suppressor genes and increasing the ability of viral DNA to transform cells and induce carcinogenesis. 29

Studies have pointed out a higher risk of high-grade cervical lesions related to the use of COCs in women with hr-HPV infection, suggesting an interaction between HPV infection and COCs with increased HPV genome expression in neoplasms of COCs users. 30 31 However, although Kjellberg et al. found an association between prolonged use of COCs and high-grade lesions, the association lost significance after considering HPV infection. 32

A systematic review published in 2003 addressed the relationship between carcinoma in situ or CC and the duration and current use of hormonal contraceptives, with special attention to hr-HPV infection. Twenty-eight eligible studies were identified, including 12,531 women with CC. Compared with women who never used COCs, the relative risks of CC in women who are users increased according to the duration of use: < 5 years, 5 to 9 years, and ≥ 10 years, respectively. Relative risks were 1.1 (95%CI = 1.1–1.2), 1.6 (95%CI = 1.4–1.7), and 2.2 (95%CI = 1.9–2.4) respectively for all women; and 0.9 (95%CI = 0.7–1.2), 1.3 (95%CI = 1.0–1.9), and 2.5 (95% CI = 1.6–3.9) for hr-HPV-positive women. Results were similar when they adjusted the data for in situ and invasive lesions, squamous cell, and adenocarcinoma, HPV status, number of sexual partners, cervical screening frequency, smoking, and barrier contraceptive use. 33 However, a recent systematic review found no consistent evidence of an association between COCs use and increased risk of pre-neoplastic lesions and CC after considering hr-HPV infection in the analysis. 34

Vessey et al. compared the incidence of CC in a prospective study with a 10-year follow-up of 6,838 COCs users and 3,154 nonhormonal intrauterine devices (IUD) users. 35 The incidence of preneoplastic lesions and CC ranged from 0.9/1,000 women per year with up to 2 years of COCs use to 2.2/1,000 women per year for those who used it for ≥ 8 years. In IUD users, there was no variation in the incidence and the rate was ∼ 1/1000 women per year. All cases of invasive cervical neoplasia occurred in the group of COCs users, 9 in women with > 6 years of use. The reduced risk of CC in diaphragm users compared with COCs users and IUD users have been previously reported, as well as the protective effect of sexual partner's vasectomy. 36 37 Moreno et al. published data from a previous IARC multicenter study analyzing CC risk in COCs users with HPV infection, including 8 case-control studies addressing CC and 2 studies on carcinoma in situ. 38 No increase in risk was observed in nonusers or those with up to 5 years of use, but there was a relative risk of 2.82 for those using for between 5 and 9 years and of 4.03 for > 10-year users. Despite these findings showing a more than twofold increase in the risk for women who have used COCs for > 5 years and with persistent hr-HPV infection, no change in contraceptive orientation was recommended when evaluating the risks and benefits of using COCs. 39

The emergence of some studies demonstrating a significant increase in risk for CC in users of COCs motivated a new publication in 2007 by an IARC advisory group. They analyzed the carcinogenic risk of COCs and combined estroprogestin therapy in postmenopausal women and finally classified COCs as carcinogenic agents. 40 According to this publication, the totality of the evidence at the time indicated that the risk of CC increased with increasing duration of COCs use. They also concluded that the risk was slightly higher for carcinoma in situ than for invasive cancer and that the relative risk seemed to decrease after use cessation. Also, in the same publication, similar results were found regardless of adjustment for the number of sexual partners, frequency of screening, smoking, and barrier contraceptive use. The possibility that the observed association was due to the higher frequency screening bias in COCs users was not excluded but was considered unlikely. 40

After this classification of COCs as carcinogenic agents by IARC due to their effect on the CC risk, a revision of 24 published epidemiological studies including data from 16,573 women with CC and 35,509 controls was performed by several researchers from the International Collaboration of Epidemiological Studies of Cervical Cancer in the same year. The results confirmed a relative risk of 1.9 for users of > than 5 years for both invasive cancer and carcinoma in situ. It also suggested that 10 years of use around the age of 20 or 30 years old would lead to an increased incidence of CC by age 50 years old from 7.3 to 8.3 per 1,000 women in less developed countries. The risk seems to decrease with use cessation, being similar that of nonusers after 10 years. 41

The World Health Organization (WHO) has also shown concern about an association between CC and the use of COCs. In 1977, a scientific group was convened to review the possible carcinogenic effect of hormonal contraceptives and identify new studies needed. In that regard, a multicenter case-control study was initiated with data from several countries, mainly developing countries. Preliminary data identified a CC risk of 1.19 for women who used COCs for up to 5 years and 1.53 for > 5-year users. 42 Final data were published in 1993, suggesting a causal relationship between the use of COCs and CC with a relative risk of 1.31 for women who used up to 4 years and a significant increase in the risk for longer-term users, reaching 2.25 for those who have used it for > 10 years. However, the risk returned to the basal status after 8 years of use cessation. In conclusion, the study suggests priority in CC screening in patients who have used COCs for > 4 years. 43 Also, Roura et al. published the results of a prospective cohort of 308,036 women recruited in the European Prospective Investigation in Cancer and Nutrition (EPIC) Study with a mean follow-up of 9 years. They counted 261 cases of CC and 804 cases of grade 3 intraepithelial neoplasia or carcinoma in situ. Duration of COCs use was associated with increased risk of grade 3 intraepithelial neoplasia/carcinoma in situ and CC, hazard ratio (HR) = 1.6 and |HR = 1.8, respectively, for 15-year users versus never-users. 44

The risk of COCs use and the development of adenocarcinoma specifically has also been investigated. A review of case-control studies for adenocarcinoma alone was published by Castellsagué et al., who found evidence that prolonged use of COCs in hr-HPV positive patients increased the risk of adenocarcinoma (OR = 4.71 for those who had > 5 years of use). 45 Also, a systematic review published in 2020 including 19 studies pointed to a higher association of COCs and cervical adenocarcinoma (1.77; 95%CI: 1.4–2.24), compared with 1.29 (95%CI: 1.18–1.42) in invasive squamous cervical cancer and 1.7 in carcinoma in situ (95%CI: 1.18–2.44). 46

In addition, an analysis of CC mortality and COCs use was addressed. A prospective study with 25-year follow-up data on 46,000 British women found a 2.5-fold increase in women using COCs, or those who had recently used them (up to 10 years) after adjusting the data for parity, social class, and smoking. 47

On the other hand, some publications found no association between COCs and CC. Syrjänen et al., analyzing screening data from a cohort study in the former Soviet Union, concluded that COCs are not an independent risk factor for intraepithelial neoplasia or hr-HPV infection. Analysis of data from COCs nonusers, users of nonhormonal methods, and COCs users showed an identical prevalence of hr-HPV infection, cytological abnormalities, and intraepithelial neoplasia histology, but with significant differences ( *p* < 0.001) on all sexual behavior variables. 48 Similar results were described by Longatto-Filho et al., analyzing data from a cohort study of over 12,000 Brazilian and Argentinian women. In this study, patients using diverse hormonal contraceptive methods (oral, injectable, patch, implant, vaginal ring, and levonorgestrel IUD) were included and no evidence was found that hormonal contraceptive use and duration of use are independent risk factors for hr-HPV infection or high-grade cervical intraepithelial neoplasia. 49 In 2017, a meta-analysis of case-control studies including 7,433 cases and 8,186 controls showed no association between COCs use and CC, although an increase was seen in the Asian population. 50 Consistent results were found in a prospective study with a follow-up of 46,022 women who were ≥ 44 years old, demonstrating that women who choose to use COCs are not exposed to increased long-term cancer risk. 51

Despite the tendency of signaling the interaction of COCs and hr-HPV infection in the emergence of cervical lesions, there are evident limitations in the standardization of studies that have aimed to define the impact of their use on cervical carcinogenesis. Retrospective analyses based on different active ingredients, distinct combinations, multiple dosages, and variables in the duration of COCs use are confounding factors that prevent more robust and definitive conclusions. There are gaps in scientific knowledge that deserve to be explored. Can distinct histological types – squamous or glandular lesions – be differently impacted by COCs use? Considering cohorts of women vaccinated against hr-HPV, will COCs users be at higher risk of developing cervical lesions? Which group of women using COCs would be more exposed to a higher risk of developing CC and its precursor lesions? Under what conditions the suspension of the COCs and encouragement of an alternative contraceptive method would be indicated?

The wide long-term use of COCs in Brazilian women requires more studies to define such risk in the Brazilian female population. At least, patients should receive information about the risks and benefits of using COCs for > 5 years, with counseling about the importance of adherence to CC screening. Also, maybe in this population of > 5-year-COC users, there should be discussions about the use of hr-HPV testing for screening.

Considering that the use of combined contraceptives increases the risk of some types of cancer and reduces others, a rigorous analysis of the overall outcome of this equation for the Brazilian female population is needed; especially because the country still has a high incidence of CC with great public health implications.

## Conclusion

Despite controversial data in the literature, several populational studies suggest a possible increase in the incidence of CC and its precursor lesions for those who have used COCs for > 5 years. Detailed studies about the impact of this increased risk in the high incidence of CC in Brazilian women who have been using COCs for a long time are still necessary to evaluate risks and benefits, as well as adequate screening coverage for CC in this specific population.
